# Cobalamin and folate status in women during early pregnancy in Bhaktapur, Nepal

**DOI:** 10.1017/jns.2021.53

**Published:** 2021-08-09

**Authors:** Catherine Schwinger, Shakun Sharma, Ram K. Chandyo, Mari Hysing, Ingrid Kvestad, Manjeswori Ulak, Suman Ranjitkar, Merina Shrestha, Laxman P. Shrestha, Adrian McCann, Per M. Ueland, Tor A. Strand

**Affiliations:** 1 Centre for Intervention Science in Maternal and Child Health, Centre for International Health, Department of Global Public Health and Primary Care, University of Bergen, Bergen, Norway; 2 Department of Child Health, Institute of Medicine, Tribhuvan University, Kathmandu, Nepal; 3 Department of Community Medicine, Kathmandu Medical College, Kathmandu, Nepal; 4 Department of Psychosocial Science, Faculty of Psychology, University of Bergen, Bergen, Norway; 5 Regional Centre for Child and Youth Mental Health and Child Welfare, NORCE Norwegian Research Centre, Bergen, Norway; 6 Bevital AS, Bergen, Norway; 7 Department of Clinical Science, University of Bergen, Bergen, Norway; 8 Department of Research, Innlandet Hospital Trust, Lillehammer, Norway

**Keywords:** Asia, Folic acid, Low-income country, Micronutrient malnutrition, Nutritional status, Vitamin B12

## Abstract

The demand for cobalamin (vitamin B12) and folate is increased during pregnancy, and deficiency during pregnancy may lead to complications and adverse outcomes. Yet, the status of these micronutrients is unknown in many populations. We assessed the concentration of cobalamin, folate and their functional biomarkers, total homocysteine (tHcy) and methylmalonic acid (MMA), in 561 pregnant women enrolled in a community-based randomised controlled trial in Bhaktapur, Nepal. Plasma concentrations of cobalamin, folate, tHcy and MMA were measured and a combined indicator of vitamin B12 status (3cB12) was calculated. We report mean or median concentrations and the prevalence of deficiency according to commonly used cut-offs, and assessed their association with indicators of socio-economic status, and maternal and dietary characteristics by linear regression. Among the women at gestational week less than 15, deficiencies of cobalamin and folate were seen in 24 and 1 %, respectively. Being a vegetarian was associated with lower plasma cobalamin, and a higher socio-economic status was associated with a better micronutrient status. We conclude that cobalamin deficiency defined by commonly used cut-offs was common in Nepalese women in early pregnancy. In contrast, folate deficiency was rare. As there is no consensus on cut-off points for vitamin B12 deficiency during pregnancy, future studies are needed to assess the potential functional consequences of these low values.

## Introduction

Cobalamin and folate, also known as vitamin B12 and vitamin B9, respectively, are crucial for rapid cell division and anabolic processes, as well as for the synthesis of DNA^(1)^. In addition, both have essential roles in the central nervous system and in the methylation cycle^(2,3)^, and deficiency in either micronutrient can lead to megaloblastic anaemia^(4,5)^. Overt deficiency and suboptimal status of these two vitamins are major public health concerns affecting a large number of people across all ages worldwide^(6–8)^.

During pregnancy and lactation, women have a higher risk of deficiency due to an increased demand for vitamin B12 and folate^(9–11)^. In a meta-analysis including fifty-seven studies from across the globe, it was estimated that one-fourth of all pregnant women were cobalamin-deficient across all trimesters of pregnancy^(9)^. In the four studies from the Indian subcontinent included in this meta-analysis, 32 % of women were defined as cobalamin-deficient in their first trimester, and 64 % in the second trimester. Both maternal vitamin B12 deficiency and folate deficiency have been associated with an increased risk of complications and poorer outcomes in pregnancy^(12–20)^ as well as long-term consequences such as impaired postnatal growth and cognitive development^(21–25)^.

Total homocysteine (tHcy) and methylmalonic acid (MMA) are functional biomarkers of vitamin B12 status, both increasing with decreasing cobalamin concentration. While MMA is a specific indicator of cobalamin, tHcy is influenced by other factors, in particular folate status^(17,26)^. A combined indicator of vitamin B12 has been proposed which includes both direct and functional biomarkers, namely cobalamin, MMA, tHcy and holotranscobalamin^(27)^.

The most direct causes of vitamin B12 and folate deficiency are related to the diet and to malabsorption^(28)^. Biologically active cobalamin is only found in animal-derived foods, while folate is most abundant in green leafy vegetables, legumes and some fruits. Therefore, vegetarians are at higher risk of a vitamin B12 deficiency^(28,29)^, and populations consuming diets low in fresh fruits, vegetables and cereals are at greater risk of folate deficiency^(30)^. A social gradient in vitamin status has also been demonstrated, with an increasing level of deficiency associated with lower socio-economic status (SES)^(29,31,32)^.

Evidence on the prevalence of vitamin B12 or folate deficiency is often derived from non-representative surveys with relatively small sample sizes. In Nepal, there is no population-based prevalence data evaluating vitamin B12 and folate status among pregnant women. However, in non-pregnant women aged 15–49 years, the Nepal National Micronutrient Status Survey 2016 reported a folate deficiency in 5 % of the women (erythrocyte folate <226⋅5 nmol/l)^(33)^. Other studies suggest a high prevalence of cobalamin deficiency in Nepal among non-pregnant^(34)^ and pregnant women^(35,36)^, while folate deficiency was uncommon^(34–36)^. However, these studies do not represent recent population data, as most of these studies were performed before 2001. In the present study, we aimed to assess the vitamin B12 and folate status using plasma concentrations of cobalamin, folate and their functional biomarkers, namely tHcy and MMA, among a community-based population of women in early pregnancy from the municipality of Bhaktapur, Nepal. We also aimed to assess investigate both direct and functional biomarkers of vitamin B12 and folate in relation to indicators of SES, and maternal and dietary characteristics.

## Methods

### Study site and population

The study site, Bhaktapur municipality and its surrounding area, lies nearly 15 km east of the capital city of Nepal, Kathmandu. Bhaktapur is an ancient and well-preserved city, settled primarily by the Newar ethnic group. According to the last census in 2011, nearly 82 000 inhabitants resided in Bhaktapur municipality. The population is a predominantly agriculture-based semi-urban community. Many households depend on small business or other daily work and services for additional income. Domestic migrant workers from nearby districts with diverse ethnic backgrounds work seasonally in carpet factories and brick industries within Bhaktapur.

The area is characterised by warm and temperate climate with a hot and wet summer, and a cold and dry winter. The average annual temperature and rainfall are 17⋅9 °C and 1583 mm, respectively^(37)^. While rice is the staple food throughout the year, inhabitants mostly consume locally grown food with vegetables and fruit intake varying in relation to the season. Intake of non-vegetarian foods is common during cultural celebrations and feasts. Factors such as availability of food, varying food prices and workload also determine the food intake of residents.

Eligible women for the study were screened from the community as well as from the study site hospital, the Siddhi Memorial Hospital for women and children, which is a non-governmental community hospital located in Bhaktapur.

### Study design and study sample

Data for the present study were gathered from the baseline assessment of an ongoing community-based, individually randomised, double-blinded placebo-controlled trial that intended to assess the effects of daily vitamin B12 supplementation from early pregnancy (<15 weeks of gestation) up to 6 months post-partum on child growth and neurodevelopment. Details of the main study have been published elsewhere^(38)^. In brief, 800 pregnant women aged 20–40 years, residing in the Bhaktapur municipality and nearby areas, not intending to leave their current settlement for at least 2 years, were targeted for inclusion in the study. Additional inclusion criteria were body mass index (BMI) between 18⋅5 and 30, not chronically ill and under treatment, and no severe anaemia. Vitamin B12 has not yet been recommended for universal use during pregnancy in Nepal, but often medications or nutrient supplements containing vitamin B12 are prescribed for pregnant women. Women were excluded if they were already taking nutrient supplements containing vitamin B12.

If they had not started taking it before enrolment, folic acid was provided to all participants during the first 2 months of pregnancy, and iron and calcium after gestational week 12, as per national guidelines from the Government of Nepal. For the present study, the results of blood sample analyses were available for the first 561 pregnant women enrolled between April 2017 and March 2020.

### Data collection

Socio-demographic information was collected at enrolment through interview. Information on education was categorised into illiterate or primary level (education up to grade 5), secondary level (grade 6–12) and Bachelor's level or above. Ethnic affiliation was grouped into Newar, Brahmin/Chhetri or Gurung/Rai/Magar/Tamang. All other ethnic groups such as Chaudhari, Madhesi, Muslims and Dalit were categorised as others. The Newar ethnic group was chosen as a reference group as it constituted the majority among our study participants (~80 %). Gestational week was estimated from the date of the first day of the last menstrual cycle (LMP method) and ascertained by ultrasound scans. Family type was defined as nuclear if only the parents and the child were living together, and joint or other, if more family members lived in the household. Parity was categorised as *primi* (having one child), and *multi* (having two or more children). The mothers were also asked about their age, as well as current smoking and alcohol consumption habits. Occupation was categorised as housewives, working in services (both private and governmental), owning a (small-scale) business, daily wage earners and others which includes those working in agriculture and without formal employment. We asked the mothers if they had or have had any pregnancy-related complaint, such as nausea/vomiting, appetite loss, abdominal pain and dizziness. We dichotomised this variable as complaints and no complaints.

Weight and height were measured using an electronic weighing scale and stadiometer (both seca, Germany). BMI was calculated as body weight (in kg) divided by the square of body height (in m).

### Blood sampling and biochemical analyses

At enrolment, i.e. before any supplement had been given, 3 ml of blood were collected into vials containing EDTA as anticoagulant for the study purpose at the field office laboratory. The blood samples were centrifuged at approximately 700 g at room temperature for 10 min; plasma was then separated and collected into three cryovials while cells were transferred separately into another cryovial. All cryovials were labelled and then stored and transported at a maximum of −72 °C in liquid nitrogen, dry ice and ultra-freezers until analysis.

Following blood draw, haemoglobin concentration was analysed immediately using HemoCue (Vedbæk, Denmark), calibrated based on manufacturer's guidelines. Concentrations of plasma cobalamin and folate were assessed by microbiological assays based on a colistin sulphate-resistant strain of *Lactobacillus leichmannii*^(39)^ and chloramphenicol-resistant strain of *Lactobacillus casei*^(40)^, respectively, adapted to a microtiter format performed by a robotic workstation. Concentrations of the functional biomarkers, plasma tHcy and MMA were analysed using gas chromatography-tandem mass spectrometry based on methylchloroformate derivatisation^(41)^. For both cobalamin and folate, the within-day coefficient of variation was 4 %, for tHcy 1 % and MMA 1–4 %. The between-day coefficient of variation was 5 % for cobalamin and folate, 2 % for tHcy and 3–8 % for MMA. Plasma concentrations of cobalamin, folate, tHcy and MMA were analysed at Bevital AS, Bergen, Norway (www.bevital.no).

### Definitions of cut-offs

The cut-off value adopted for low cobalamin was <150 pmol/l^(42)^ and for low folate <10 nmol/l^(43)^. In addition, we report low cobalamin with a cut-off <250 pmol/l as suggested to indicate inadequacy^(44)^. For high tHcy, we used the cut-off value of >10 μmol/l^(45)^, and for high MMA >0⋅26 μmol/l^(35,42,46)^. For anaemia, we report both the commonly used cut-off value of 11 g/dl for haemoglobin, as well as the altitude-adjusted (1400 m) cut-off value of <11⋅3 g/dl^(47,48)^.

We calculated a combined indicator of vitamin B12 status (3cB12) according to Fedosov *et al.*^(27)^, as log(B12/Hcy*MMA), adjusted with the folate and age factor given by Fedosov. For the purpose of the present study, we categorised 3cB12 as <−0⋅5 (vitamin B12 deficiency) and ≥−0⋅5 (vitamin B12 adequacy).

### Statistical analyses and justification of sample size

As we used data from an existing study, the sample size was set at 561 women. With this sample size, we could identify the prevalence of a certain nutrient deficiency with a 95 % confidence interval (CI) of not more than ±4 percentage points around a given estimate. In this calculation, we assumed a prevalence of 50 % as this is the most conservative approach; a higher or lower prevalence would result in a higher precision of the estimate.

All data were double entered in a local database and discrepancies and completeness were checked by the data entry supervisor. Data were analysed using Stata, version 16^(49)^. For describing the study sample characteristics, we report mean and standard deviation (sd), median and interquartile range (IQR), or percentage, as appropriate.

We used boxplots to describe the distribution of cobalamin, folate, tHcy, MMA, the combined indicator of cobalamin (3cB12) and haemoglobin, where a red line represents the respective cut-off value in each boxplot. We depicted the relation between cobalamin and its functional biomarkers (tHcy and MMA) using fractional-polynomial prediction plots.

For all indicators used, we built separate bivariable linear regression models with all variables included in Table 1 as independent variables. Folate, tHcy and MMA were not normally distributed (skewness >2) and therefore log-transformed. For the multivariable linear model, we followed the purposeful selection procedure described by Hosmer *et al.*^(50)^. If the variables were significantly associated with the outcome in the bivariable models on the basis of a *P*-value < 0⋅2, they were entered into a multivariable linear regression model simultaneously. Then, we removed the variable with the highest *P*-value. This step was repeated until all significant variables (*P* < 0⋅05) were retained in the saturated model. All variables not in the saturated model were then entered again, one by one, and retained if they now were significant (*P* < 0⋅05).

**Table 1.** Socio-economic, maternal and dietary characteristics of 561 pregnant women (<15 weeks of gestation) living in Bhaktapur, Nepal

Characteristics	Mean (sd)	Number (%)
Age (years)	27⋅5 (3⋅8)	
Body Mass Index (kg/m^2^)	23⋅7 (3⋅0)	
Vegetarian mothers^a^		7 (1⋅7)
Gestational week^b^	11 (2⋅9)	
Parity (%)		
Primi		237 (42⋅2)
Second gravida or more		324 (57⋅8)
Educational level (years)		
Illiterate or primary level (below 5th grade)		44 (7⋅8)
Secondary level (6th–12th grade)		392 (69⋅9)
Bachelor's level and above		125 (22⋅3)
Occupation of mother		
Housewife		182 (32⋅4)
Services		165 (29⋅4)
Business		116 (20⋅7)
Daily wage		81 (14⋅5)
Others		17 (3⋅0)
Average monthly household income (Nepali rupees), *median* (*IQR*)	30 000 (20 000–50 000)	
Family type		
Nuclear		192 (34⋅2)
Joint and others		369 (65⋅8)
Household size, *median* (*IQR*)	5 (3–6)	
Female head of household		73 (13)
Ethnicity of household		
Newar		440 (78⋅4)
Brahmin/Chhetri		52 (9⋅3)
Gurung/Rai/Magar/Tamang		54 (9⋅6)
Others		15 (2⋅7)
Current Smoker		2 (0⋅4)
Alcohol consumption^c^		
No alcohol		309 (55⋅1)
Once a month or less		208 (37⋅1)
Few times a month		38 (6⋅8)
More than once a week		6 (1⋅0)

a *N* 406, vegetarian was defined as not eating meat and eggs.

b Assessed by the last menstrual period (LMP) method and confirmed by ultrasound scan.

c Usually local rice beer.

### Ethical considerations

The main study was conducted according to the guidelines laid down in the Declaration of Helsinki and all procedures involving human subjects were approved by the Nepal Health Research Council (NHRC, #253/2016) and the Regional Committee for Medical and Health Research Ethics of Western Norway (2016/1620/REK vest). The study is also registered at clinicaltrials.gov (NCT03071666), and under the Universal Trial Number (U1111-1183-4093). At the implementation site in Bhaktapur, informed written consent was obtained from eligible and willing pregnant women prior to enrolment.

## Results

### Characteristics of the study cohort

The characteristics of the 561 women included in the present study are shown in Table 1. The women had a mean age of 27⋅5 years (±3⋅8 years), a mean BMI of 23⋅7 (±3⋅0) and a mean gestational week of 11 (±2⋅9 weeks). Less than 2 % of the enrolled women were vegetarian, and less than half of the vegetarian women reported eating eggs. The current pregnancy was the first for 42 % of the women. Twenty-two percent of the women had completed education up to Bachelor's level or above, while nearly 8 % of the mothers were either illiterate or had ended their education before 6th grade.

### Medications and health-related complaints

At the time of study enrolment, 69 % of the women had already started folic acid supplementation before the study. Out of 253 participants enrolled at or after gestation week 12, 86 (34 %) had already started iron supplementation as per national guidelines. Approximately 12 % of the women were taking over-the-counter multivitamins or nutrient supplements without vitamin B12 at the time of enrolment. One in five of the participants were taking other medications, such as anti-emetic drugs. Less than 1 % of the women were taking antibiotics at the time of enrolment. Plasma cobalamin concentrations did not differ between those who had started folic acid, iron or multivitamin supplementation at the time of enrolment. However, the mean plasma folate concentration was higher among those taking multivitamins compared with those who were not (78 nmol/l *v*. 62 nmol/l; *t*-test, *P* < 0⋅05).

Approximately one in three women reported pregnancy-related complaints. Nausea and vomiting were the most common complaints (reported by 26 % of all the mothers), followed by loss of appetite (6 %), lower abdominal pain (3 %) and dizziness/vertigo (1 %).

### Plasma biomarker profile

**Fig. 1.**
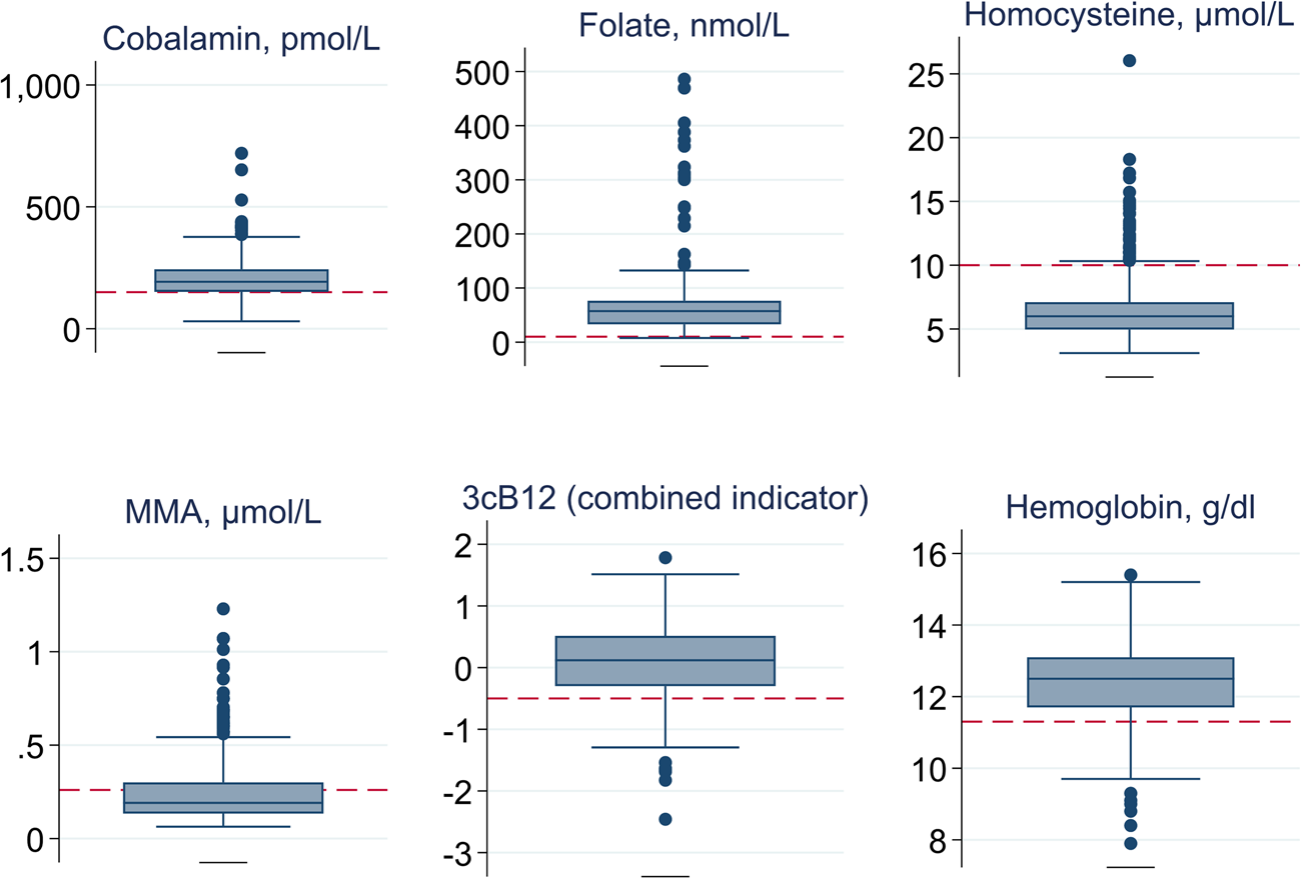
Distribution of plasma cobalamin, folate, total homocysteine (tHcy), methylmalonic acid (MMA), a combined indicator of vitamin B12 status (3cB12) and haemoglobin among 561 pregnant women (<15 weeks of gestation) from Bhaktapur, Nepal. Boxplot graphs are used to indicate the median (middle line in the box), the IQR (box around the 25th and 75th percentile), the upper and lower extreme values (values within 1⋅5 times the IQR; the whiskers), as well as outliers (dots outside the whiskers). Reference cut-offs used in the present study are added to these graphs (red dashed lines).

The distribution for plasma concentrations of cobalamin, folate, tHcy, MMA and haemoglobin of all study participants (*n* 561) is illustrated in Fig. 1, and mean plasma concentrations are presented in Table 2. Approximately 24 % of the pregnant women were found to be cobalamin-deficient (plasma cobalamin <150 pmol/l), while the combined indicator (3cB12) indicated a vitamin B12 deficiency among 16 % of the women. One percent of the participants were folate-deficient, and 16 % were anaemic.

**Table 2.** Mean concentrations of plasma cobalamin, folate and their functional biomarkers, and haemoglobin for 561 pregnant women (<15 weeks of gestation) living in Bhaktapur, Nepal

Micronutrients	Mean concentration (sd)	Cut-off	Prevalence of suboptimal concentration, % (*n*)^a^
Cobalamin (pmol/l)	204⋅5 (78⋅5)	<150	24⋅4 (137)
		<250	77⋅3 (434)
Folate (nmol/l)	63⋅6 (53⋅8)	<10	<1 (2)
Total homocysteine (μmol/l)	6⋅4 (2⋅4)	>10	7⋅7 (43)
Methylmalonic acid (μmol/l)	0⋅24 (0⋅16)	>0⋅26	32⋅8 (184)
Combined vitamin B12 indicator (3cB12)	0⋅10 (0⋅60)	<−0⋅5	16⋅0 (90)
Haemoglobin (g/dl)	12⋅4 (1⋅1)	<11⋅3	16⋅0 (90)
		<11	10⋅5 (59)

a Suboptimal concentration is defined as value above the chosen cut-off for total homocysteine, methylmalonic acid; for all other indicators, it is a value below the indicated cut-off.

### Association of cobalamin with functional biomarkers

**Fig. 2.**
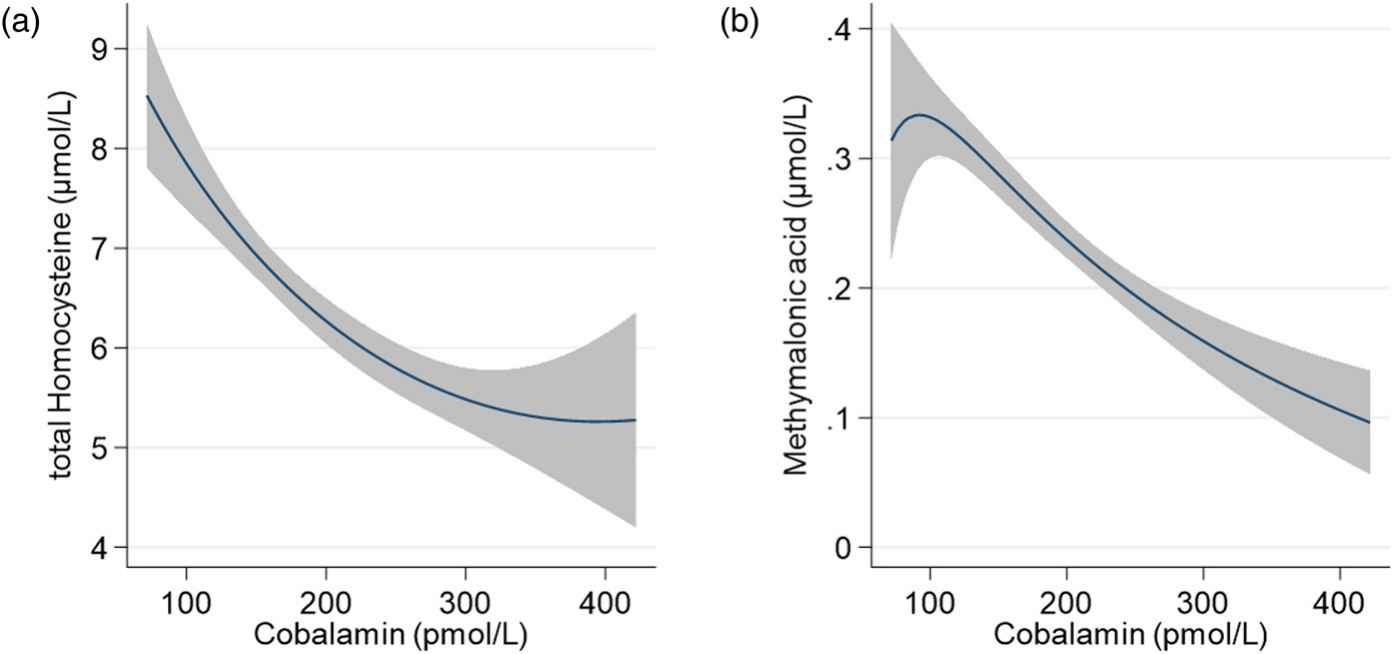
Association of cobalamin with (a) total homocysteine (tHcy) and (b) methylmalonic acid (MMA) among 561 pregnant women (<15 weeks of gestation) from Bhaktapur, Nepal, using fractional-polynomial prediction plots. The line indicates the predicted mean, and the grey area depicts the 95 % confidence interval. Values for cobalamin are restricted to the 1st to 99th centile.

Fig. 2 illustrates the relation between plasma cobalamin *v*. tHcy (Panel a) and cobalamin *v*. MMA (Panel b). Both functional markers show a negative association with plasma cobalamin which appears to be close to linear.

### Factors associated with cobalamin and folate

**Fig. 3.**
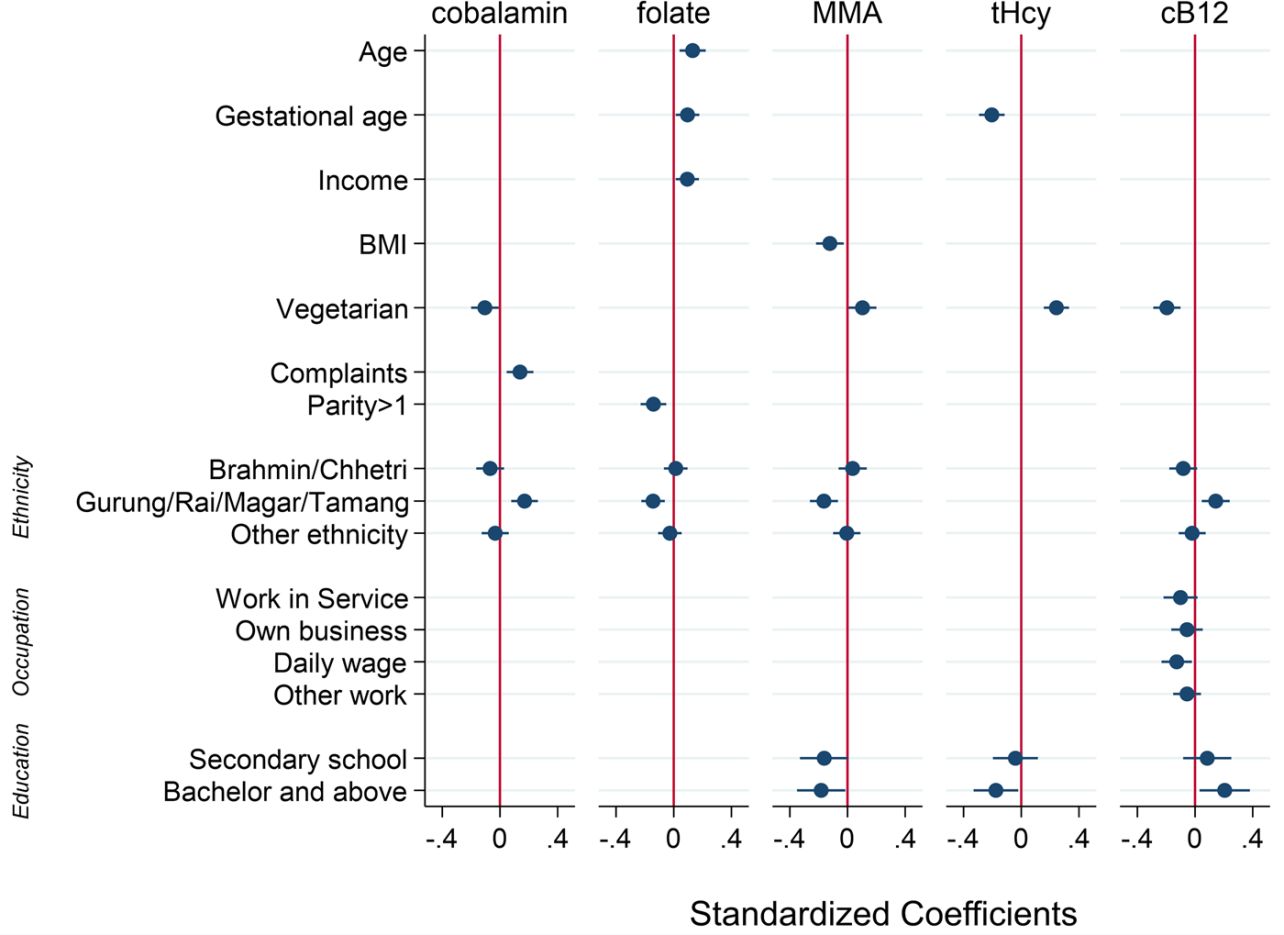
Factors associated with plasma concentrations of cobalamin, folate, total homocysteine (tHcy), methylmalonic acid (MMA) and a combined indicator of vitamin B12 status (3cB12) among 561 pregnant women (<15 weeks of gestation) from Bhaktapur, Nepal. Displayed are standardised coefficients (beta) with 95 % confidence intervals from multivariable linear regression models. Reference categories are non-vegetarian, no health-related complaints, the ethnic group Newar, parity = 1, educational level = illiterate or primary level, and the occupational group of housewives.

All results from the linear regression analyses are presented in Supplementary Tables S1–S5 of the supplementary material. Fig. 3 illustrates the standardised coefficients (beta) of the variables retained in the final multivariable models.

In the final multivariable model, being a vegetarian was associated with a lower cobalamin concentration (adjusted coefficient −66⋅6; 95 % CI −123⋅9, −9⋅37). The same association was observed for the functional markers, both of which were on average higher in the vegetarian group compared with those eating meat (MMA: 0⋅48; 95 % CI 0⋅04, 0⋅92; tHcy: 0⋅57; 95 % CI 0⋅35, 0⋅79). Having reported pregnancy-related complaints was associated with a higher cobalamin concentration (22⋅5; 95 % CI 7⋅39, 37⋅6). Being affiliated with the ethnic grouping of Gurung/Rai/Magar/Tamang was associated with higher cobalamin compared with the Newar ethnic group (54⋅8; 95 % CI 27⋅4, 82⋅2). Although significant in the bivariable analyses, maternal age, BMI, gestational week, maternal educational level and the frequency of alcohol consumption were no longer significantly associated with cobalamin in the final model (*P* > 0⋅05).

For folate, being affiliated with the Gurung/Rai/Magar/Tamang ethnic group was associated with a lower plasma concentration (−0⋅31; 95 % CI −0⋅49, −0⋅13). Age (0⋅02; 95 % CI 0⋅006, 0⋅03), gestational week (0⋅02; 95 % CI 0⋅002, 0⋅04) and monthly income (0⋅001; 95 % CI 0⋅0001, 0⋅002) were positively associated with folate status, while having two or more children was associated with a lower mean folate concentration compared with having only one child (−0⋅18; 95 % CI −0⋅30, −0⋅06). Although significant in the bivariable analyses, maternal educational level, occupation group, family type and frequency of alcohol consumption were no longer significantly associated with folate.

## Discussion

In this cross-sectional study investigating cobalamin and folate status among pregnant Nepalese women of gestational age 5–15 weeks, 24 % women were cobalamin-deficient, and 1 % were folate-deficient according to commonly used cut-offs for deficiency.

The status of vitamin B12 among pregnant women in their first trimester in the Bhaktapur district, Nepal, has not previously been assessed in a community-based setting. Among two earlier studies suggesting a high prevalence of cobalamin deficiency among pregnant women, one was hospital based^(35)^, and another was based in a district in the Southern plains of Nepal^(36)^. Our study suggests that one in four pregnant women in their first trimester was vitamin B12-deficient when using plasma cobalamin as the sole marker for status. In resource-poor settings, diets are often plant-based and low in animal-derived foods due to factors such as high cost, lack of accessibility, availability and impact of cultural or religious beliefs^(28)^. As the main source of vitamin B12 is animal-derived food items, populations in resource-poor settings are especially vulnerable to vitamin B12 deficiency. In similar settings, seasonal shortages of vegetables and fruits, or replacement of these food with low-nutrient dense foods such as polished white rice due to low economic status, has been described as a cause of short-term folate deficiency^(9)^. Typically, such deficiency can be relieved quickly without any adverse health effects; however, for women in their first trimester, even short-term shortages of food items high in folate have been shown to severely affect the foetus^(51)^. In the hills and mountains of Nepal, common household diets were mostly deficient in vitamin B12 and calcium^(52)^. This could explain the relatively high prevalence of cobalamin deficiency shown by our study.

Only 2 % of the participants reported to be vegetarian. In agreement with previous studies^(29,53–55)^, these pregnant women were found more likely to have poor vitamin B12 status. However, our findings demonstrate that cobalamin deficiency was not limited to vegetarian women. It has been suggested that even among individuals who do not identify as being vegetarian, consumption of animal-derived food/meat might be limited due to affordability and religious beliefs^(34,54,56)^.

Our findings suggest that less than 1 % of the pregnant women were folate-deficient. This is in line with other studies showing that folate deficiency was not common in Nepal, neither in adults^(34,35,36)^ nor in children^(31,53,57,58)^. The adequate folate status may be attributed to ongoing folic acid supplementation during pregnancy as part of the national nutrition programme, as well as a generally high consumption of foods rich in folate, such as green leafy vegetables^(59)^. Although food intake varies according to the season in this population, we did not see a clear seasonal trend in folate concentration according to timing of the blood draw (data not shown). In our study, two out of every three participating pregnant women had already begun folic acid supplementation as per national guidelines before the time of enrolment. For some women, high plasma folate concentration may be explained by the folate/methyl-trap hypothesis related to vitamin B12 deficiency, intake of oral supplements shortly before blood sampling, or high consumption of foods rich in folate^(44,60)^.

In addition to the direct measures of cobalamin status, we also assessed the functional biomarkers tHcy and MMA^(61–65)^. There was an inverse association between plasma cobalamin concentration and both tHcy and MMA concentration, which has also been described previously^(61)^. In our sample, the prevalence of abnormal values was lower for tHcy (7 %) and higher for MMA (33 %) compared to cobalamin (24 %). Circulating tHcy levels are dependent on the availability of both cobalamin and folate, while MMA is not affected by folate status^(61)^. Folate deficiency was rare in our study participants. In addition, other studies undertaken in Nepal and India have demonstrated that tHcy was not associated with dietary intake or vitamin B12 supplementation in pregnant women^(21,66)^. Notably however, in the Indian study, vitamin B12 supplementation of the mothers reduced plasma tHcy concentrations in their infants at 6 weeks after birth^(66)^. This further supports evidence suggesting that tHcy may not be a good indicator of vitamin B12 status during pregnancy. MMA is considered a more specific marker of cobalamin function^(46,67)^ and may help explain the differences in prevalence that we observed. However, MMA might have been altered to a larger extend by intestinal bacterial overgrowth and infection^(44)^. We did not have data to support this. To increase the precision of diagnosing cobalamin deficiency, Fedosov *et al.*^(27)^ suggested the use of an indicator combining several biomarkers of vitamin B12, with additional adjustment for age and folate status. Using their formula for the combined cobalamin indicator (3cB12), the prevalence of cobalamin deficiency was 16 % in our study sample. A lower prevalence of low vitamin B12 status measured by the 3cB12 indicator (47 %) compared with plasma vitamin B12 (<148 pmol/l) (63 %) has also been reported in a group of pregnant women at 12 weeks of gestations in India^(68)^. However, to our knowledge, the use of the combined indicator is yet to be validated among pregnant women and reasons for the discrepancy between indicators remain unclear.

A deficiency of cobalamin and folate may result in anaemia, as both are required for erythropoiesis^(4,5)^. In our study, 16 % of the pregnant women were anaemic according to a haemoglobin cut-off at 11⋅3 g/dl (altitude-adjusted) and 10⋅5 % with a cut-off at 11 g/dl. The Nepal Micronutrient Status Survey 2016 suggested a higher prevalence of anaemia among pregnant women of nearly 27 % when using 11 g/dl of haemoglobin as cut-off^(33)^. The higher prevalence in the national sample could be attributed to differences in gestational age, maternal age and sampling location. In the present study, cobalamin deficiency was not associated with anaemia, in concurrence with an earlier study performed in the same setting among non-pregnant women^(34)^.

In our study, pregnancy-related complaints were associated with a higher mean concentration of plasma cobalamin. The interpretation of this observation is hindered by the non-specific categorisation of all complaints into a single group. Nonetheless, the finding appears somewhat counterintuitive, as lower food intake and subsequently lower cobalamin status would be expected to occur as a result of pregnancy-related complaints such as vomiting and nausea. However, Nepali women could have taken ready-made and fortified foods to compensate the potential nutrient loss due to hyperemesis gravidum. We did not adjust the analysis for dietary intake and thus, cannot confirm this.

Higher SES was associated with better cobalamin and folate status in our analysis; this is in line with findings from a study of infants living in the same area^(31)^ and has been repeatedly documented in other studies^(28)^. Diets of populations with a low SES are typically low in vitamin B12, due to higher prices and lower availability of animal-derived foods. These populations are also characterised by a higher disease burden, which can increase nutrient requirements and impair the nutrient absorption^(28)^.

Although Smith^(69)^ states that differences in food intake might better be described by means of income than cultural restrictions, in our study, ethnic affiliation was consistently associated with vitamin B12 and folate status, even when other indicators of SES were included in the regression models. This could indicate that in addition to differences in SES and thus economic opportunities and access to health care, cultural practices which indicate a more varied diet and/or a more frequent consumption of non-vegetarian diets could be important.

### Strengths and limitations

The key strength of the present study is the comprehensive characterisation of a large community-based cohort of women sampled in early pregnancy. In addition, standard procedures were followed for blood sample collection, storage and transfer, ensuring optimal cold chain, and state-of-the-art biochemical methods were used to estimate biomarker concentrations. During pregnancy, cobalamin and folate requirements in the body increase; however, unfortunately, we did not have data on dietary intake at the time point of enrolment. To our knowledge, there are no specific, universally accepted cut-off values for markers of vitamin B12 in early pregnancy. It has been shown that concentrations of B12 biomarkers decline during pregnancy, among others due to hemodilution. However, different cut-off during pregnancy have not been agreed upon, and cut-offs for the general population are commonly used. This is seen in a recent meta-analyses including twelve studies during the first trimester, where eight of the studies used cut-offs between 148 and 156 pmol/l^(9)^. The increase in plasma volume during pregnancy is not expected to start before 6–10 weeks^(70)^, and thus, the effect of hemodilution on the prevalence of low B12 in participants of the present study, measured on average at 11 weeks of gestation, is expected to be minimal.

## Conclusion

The prevalence of cobalamin deficiency in our study population of women in early pregnancy living in Bhaktapur, Nepal, was 24 %. In contrast, folate deficiency was rare. In light of the high levels of cobalamin deficiency observed in the present study, future studies are needed to assess the potential functional consequences of suboptimal maternal cobalamin status such as complications and adverse outcomes in pregnancy as well as long-term outcomes such as impaired postnatal growth and cognitive development.

## Supplementary material

For supplementary material accompanying this paper visit http://dx.doi.org/10.1017/jns.2021.53. click here to view supplementary material

## References

[1] van de Rest O, van Hooijdonk LW, Doets E, (2012) B vitamins and *n*-3 fatty acids for brain development and function: review of human studies. Ann Nutr Metab 60, 272–292. 22678093 10.1159/000337945

[2] Mahmood L (2014) The metabolic processes of folic acid and vitamin B12 deficiency. J Health Res Rev 1, 5.

[3] Varela-Moreiras G, Murphy MM & Scott JM (2009) Cobalamin, folic acid, and homocysteine. Nutr Rev 67, S69–S72. 19453682 10.1111/j.1753-4887.2009.00163.x

[4] Koury MJ & Ponka P (2004) New insights into erythropoiesis: the roles of folate, vitamin B12, and iron. Annu Rev Nutr 24, 105–131. 15189115 10.1146/annurev.nutr.24.012003.132306

[5] Stabler SP (2020) Alterations in sulfur amino acids as biomarkers of disease. J Nutr 150, 2532S–2537S. 33000156 10.1093/jn/nxaa118

[6] de Benoist B (2008) Conclusions of a WHO Technical Consultation on folate and vitamin B12 deficiencies. Food Nutr Bull 29, S238–S244. 18709899 10.1177/15648265080292S129

[7] McLean E, de Benoist B & Allen LH (2008) Review of the magnitude of folate and vitamin B12 deficiencies worldwide. Food Nutr Bull 29, S38–S51. 18709880 10.1177/15648265080292S107

[8] Smith AD, Warren MJ, Refsum H (2018) Vitamin B12. In Advances in Food and Nutrition Research, vol. 83, pp. 215–279. Cambridge, MA: Elsevier. 29477223 10.1016/bs.afnr.2017.11.005

[9] Sukumar N, Rafnsson SB, Kandala N-B, (2016) Prevalence of vitamin B-12 insufficiency during pregnancy and its effect on offspring birth weight: a systematic review and meta-analysis. Am J Clin Nutr 103, 1232–1251. 27076577 10.3945/ajcn.115.123083

[10] Casterline JE, Allen LH & Ruel MT (1997) Vitamin B-12 deficiency is very prevalent in lactating Guatemalan women and their infants at three months postpartum. J Nutr 127, 1966–1972. 9311952 10.1093/jn/127.10.1966

[11] Smith AM, Picciano MF & Deering RH (1983) Folate supplementation during lactation: maternal folate status, human milk folate content, and their relationship to infant folate status. J Pediatr Gastroenterol Nutr 2, 622–628. 6685760

[12] Liu C, Liu C, Wang Q, (2018) Supplementation of folic acid in pregnancy and the risk of preeclampsia and gestational hypertension: a meta-analysis. Arch Gynecol Obstet 298, 697–704. 29978414 10.1007/s00404-018-4823-4 PMC6153594

[13] Kharb S, Aggarwal D, Bala J, (2016) Evaluation of homocysteine, vitamin B12 and folic acid levels during all the trimesters in pregnant and preeclamptic womens. Curr Hypertens Rev 12, 234–238. 27748186 10.2174/1573402112666161010151632

[14] Chen LW, Lim AL, Colega M, (2015) Maternal folate status, but not that of vitamins B-12 or B-6, is associated with gestational age and preterm birth risk in a multiethnic Asian population. J Nutr 145, 113–120. 25527665 10.3945/jn.114.196352

[15] Muthayya S, Kurpad A, Duggan C, (2006) Low maternal vitamin B 12 status is associated with intrauterine growth retardation in urban South Indians. Eur J Clin Nutr 60, 791. 16404414 10.1038/sj.ejcn.1602383

[16] Dwarkanath P, Barzilay JR, Thomas T, (2013) High folate and low vitamin B-12 intakes during pregnancy are associated with small-for-gestational age infants in South Indian women: a prospective observational cohort study. Am J Clin Nutr 98, 1450–1458. 24108785 10.3945/ajcn.112.056382

[17] Finkelstein JL, Layden AJ & Stover PJ (2015) Vitamin B-12 and perinatal health. Adv Nutr 6, 552–563. 26374177 10.3945/an.115.008201 PMC4561829

[18] George L, Mills JL, Johansson AL, (2002) Plasma folate levels and risk of spontaneous abortion. JAMA 288, 1867–1873. 12377085 10.1001/jama.288.15.1867

[19] Nelen WL, Blom HJ, Steegers EA, (2000) Hyperhomocysteinemia and recurrent early pregnancy loss: a meta-analysis. Fertil Steril 74, 1196–1199. 11119750 10.1016/s0015-0282(00)01595-8

[20] Mobasheri E, Keshtkar A & Golalipour M-J (2010) Maternal folate and vitamin B12 status and neural tube defects in Northern Iran: a case control study. Iranian J Pediatr 20, 167. PMC3446032 23056699

[21] Strand TA, Ulak M, Kvestad I, (2018) Maternal and infant vitamin B12 status during infancy predict linear growth at 5 years. Pediatr Res 84, 611. 29967525 10.1038/s41390-018-0072-2

[22] Strand TA, Taneja S, Kumar T, (2015) Vitamin B-12, folic acid, and growth in 6-to 30-month-old children: a randomized controlled trial. Pediatrics 135, e918–e926. 25802345 10.1542/peds.2014-1848

[23] Thomas S, Thomas T, Bosch RJ, (2019) Effect of maternal vitamin b12 supplementation on cognitive outcomes in South Indian children: a randomized controlled clinical trial. Matern Child Health J 23, 155–163. 30003521 10.1007/s10995-018-2605-z

[24] Srinivasan K, Thomas T, Kapanee ARM (2017) Effects of maternal vitamin B12 supplementation on early infant neurocognitive outcomes: a randomized controlled clinical trial. Matern Child Nutr 13, e12325. 10.1111/mcn.12325 PMC6090548 27356547

[25] Molloy AM, Kirke PN, Brody LC, (2008) Effects of folate and vitamin B12 deficiencies during pregnancy on fetal, infant, and child development. Food Nutr Bull 29, S101–S111. 18709885 10.1177/15648265080292S114

[26] Stabler SP (2013) Vitamin B12 deficiency. N Engl J Med 368, 2041–2042. 10.1056/NEJMc1304350 23697526

[27] Fedosov SN, Brito A, Miller JW, (2015) Combined indicator of vitamin B12 status: modification for missing biomarkers and folate status and recommendations for revised cut-points. Clin Chem Lab Med 53, 1215–1225. 25720072 10.1515/cclm-2014-0818

[28] Allen LH (2008) Causes of vitamin B12 and folate deficiency. Food Nutr Bull 29, S20–S34. 18709879 10.1177/15648265080292S105

[29] Villamor E, Mora-Plazas M, Forero Y, (2008) Vitamin B-12 status is associated with socioeconomic level and adherence to an animal food dietary pattern in Colombian school children. J Nutr 138, 1391–1398. 18567766 10.1093/jn/138.7.1391

[30] Carmel R (2006) Folic acid. In Modern Nutrition in Health and Disease, 11th ed., pp. 470–481 [M Shils, M Shike, A Ross, B Caballero and RJ Cousins, editors]. Philadelphia: Lippincott Williams & Wilkins.

[31] Chandyo RK, Ulak M, Kvestad I, (2018) Cobalamin and folate status among breastfed infants in Bhaktapur, Nepal. Nutrients 10, 639. 10.3390/nu10050639 PMC5986518 29783689

[32] Wong AY, Chan EW, Chui CS, (2014) The phenomenon of micronutrient deficiency among children in China: a systematic review of the literature. Public Health Nutr 17, 2605–2618. 25248458 10.1017/S1368980013002978 PMC10282226

[33] Ministry of Health and Population Nepal, New ERA, UNICEF, (2018) Nepal National Micronutrient Status Survey Report 2016. Kathmandu: Ministry of Health and Population, Nepal.

[34] Chandyo R, Ulak M, Sommerfelt H, (2016) Nutritional intake and status of cobalamin and folate among non-pregnant women of reproductive age in Bhaktapur, Nepal. Nutrients 8, 375. 10.3390/nu8060375 PMC4924216 27338469

[35] Bondevik G, Schneede J, Refsum H, (2001) Homocysteine and methylmalonic acid levels in pregnant Nepali women. Should cobalamin supplementation be considered? Eur J Clin Nutr 55, 856. 11593347 10.1038/sj.ejcn.1601236

[36] Jiang T, Christian P, Khatry SK, (2005) Micronutrient deficiencies in early pregnancy are common, concurrent, and vary by season among rural Nepali pregnant women. J Nutr 135, 1106–1112. 15867289 10.1093/jn/135.5.1106

[37] Climate-Data.org (2020) Bhaktapur climate. https://en.climate-data.org/asia/nepal/central-development-region/bhaktapur-764428/.

[38] Chandyo RK, Ulak M, Kvestad I, (2017) The effects of vitamin B12 supplementation in pregnancy and postpartum on growth and neurodevelopment in early childhood: study protocol for a randomized placebo controlled trial. BMJ Open 7, e016434. 10.1136/bmjopen-2017-016434 PMC5634456 28851784

[39] Kelleher BP & Broin SD (1991) Microbiological assay for vitamin B12 performed in 96-well microtitre plates. J Clin Pathol 44, 592–595. 1856292 10.1136/jcp.44.7.592 PMC496801

[40] Molloy AM & Scott JM (1997) Microbiological assay for serum, plasma, and red cell folate using cryopreserved, microtiter plate method. Methods Enzymol 281, 43–53. 9250965 10.1016/s0076-6879(97)81007-5

[41] Midttun O, McCann A, Aarseth O, (2016) Combined measurement of 6 fat-soluble vitamins and 26 water-soluble functional vitamin markers and amino acids in 50 μL of serum or plasma by high-throughput mass spectrometry. Anal Chem 88, 10427–10436. 27715010 10.1021/acs.analchem.6b02325

[42] Refsum H, Yajnik CS, Gadkari M, (2001) Hyperhomocysteinemia and elevated methylmalonic acid indicate a high prevalence of cobalamin deficiency in Asian Indians. Am J Clin Nutr 74, 233–241. 11470726 10.1093/ajcn/74.2.233

[43] WHO (2015) Serum and Red Blood Cell Folate Concentrations for Assessing Folate status in Populations. Geneva: World Health Organization.

[44] Allen LH, Miller JW, de Groot L, (2018) Biomarkers of nutrition for development (BOND): vitamin B-12 review. J Nutr 148, 1995S–2027S. 30500928 10.1093/jn/nxy201 PMC6297555

[45] Aparicio-Ugarriza R, Palacios G, Alder M, (2015) A review of the cut-off points for the diagnosis of vitamin B12 deficiency in the general population. Clin Chem Lab Med (CCLM) 53, 1149–1159. 25470607 10.1515/cclm-2014-0784

[46] Bolann BJ, Solli JD, Schneede J, (2000) Evaluation of indicators of cobalamin deficiency defined as cobalamin-induced reduction in increased serum methylmalonic acid. Clin Chem 46, 1744–1750. 11067808

[47] CDC (1989) CDC criteria for anemia in children and childbearing-aged women. Morbid Mortal Weekly Rep 38, 400. 2542755

[48] Croft TN, Marshall AMJ, Allen CK (2018) Anemia Status. Guide to DHS Statistics. https://dhsprogram.com/data/Guide-to-DHS-Statistics/index.htm#t=Anemia_Status.htm.

[49] StataCorp (2019) Stata Statistical Software: Release 16. College Station, TX: StataCorp LLC.

[50] Hosmer DW, Lemeshow S & Sturdivant RX (2013) Applied Logistic Regression. New York, USA: John Wiley & Sons, Incorporated.

[51] Dugdale AE (2006) Predicting iron and folate deficiency anaemias from standard blood testing: the mechanism and implications for clinical medicine and public health in developing countries. Theor Biol Med Model 3, 34. 17029621 10.1186/1742-4682-3-34 PMC1626451

[52] Biehl E, Klemm RD, Manohar S, (2016) What does it cost to improve household diets in Nepal? Using the cost of the diet method to model lowest cost dietary changes. Food Nutr Bull 37, 247–260. 27378799 10.1177/0379572116657267

[53] Ng'eno BN, Perrine CG, Whitehead RD, (2017) High prevalence of vitamin B12 deficiency and no folate deficiency in young children in Nepal. Nutrients 9, 72. 10.3390/nu9010072 PMC5295116 28106733

[54] Yajnik CS, Deshpande SS, Lubree HG, (2006) Vitamin B12 deficiency and hyperhomocysteinemia in rural and urban Indians. J Assoc Physicians India 54, 775–782. 17214273

[55] Herrmann W, Schorr H, Obeid R, (2003) Vitamin B-12 status, particularly holotranscobalamin II and methylmalonic acid concentrations, and hyperhomocysteinemia in vegetarians. Am J Clin Nutr 78, 131–136. 12816782 10.1093/ajcn/78.1.131

[56] Antony AC (2003) Vegetarianism and vitamin B-12 (cobalamin) deficiency. Am J Clin Nutr 78, 3–6. 12816765 10.1093/ajcn/78.1.3

[57] Ulak M, Chandyo RK, Thorne-Lyman AL, (2016) Vitamin status among breastfed infants in Bhaktapur, Nepal. Nutrients 8, 149. 27005657 10.3390/nu8030149 PMC4808878

[58] Ulak M, Chandyo RK, Adhikari RK, (2014) Cobalamin and folate status in 6 to 35 months old children presenting with acute diarrhea in Bhaktapur, Nepal. PLoS ONE 9, e90079. 24594935 10.1371/journal.pone.0090079 PMC3940712

[59] Ministry of Health Nepal (2017) Nepal Demographic and Health Survey 2016. Kathmandu: Ministry of Health, Nepal.

[60] Paul L & Selhub J (2017) Interaction between excess folate and low vitamin B12 status. Mol Aspects Med 53, 43–47. 27876554 10.1016/j.mam.2016.11.004

[61] Hannibal L, Lysne V, Bjørke-Monsen A-L, (2016) Biomarkers and algorithms for the diagnosis of vitamin B12 deficiency. Front Mol Biosci 3, 27. 27446930 10.3389/fmolb.2016.00027 PMC4921487

[62] Allen RH, Stabler SP, Savage DG, (1993) Metabolic abnormalities in cobalamin (vitamin B12) and folate deficiency. FASEB J 7, 1344–1353. 7901104 10.1096/fasebj.7.14.7901104

[63] Monsen A-LB, Refsum H, Markestad T, (2003) Cobalamin status and its biochemical markers methylmalonic acid and homocysteine in different age groups from 4 days to 19 years. Clin Chem 49, 2067–2075. 14633879 10.1373/clinchem.2003.019869

[64] Graham I (1999) Homocysteine in health and disease. Ann Intern Med 131, 387–388. 10475892 10.7326/0003-4819-131-5-199909070-00010

[65] Snow CF (1999) Laboratory diagnosis of vitamin B12 and folate deficiency: a guide for the primary care physician. Arch Intern Med 159, 1289–1298. 10386505 10.1001/archinte.159.12.1289

[66] Duggan C, Srinivasan K, Thomas T, (2014) Vitamin B-12 supplementation during pregnancy and early lactation increases maternal, breast milk, and infant measures of vitamin B-12 status. J Nutr 144, 758–764. 24598885 10.3945/jn.113.187278 PMC3985831

[67] Clarke R, Refsum H, Birks J, (2003) Screening for vitamin B-12 and folate deficiency in older persons. Am J Clin Nutr 77, 1241–1247. 12716678 10.1093/ajcn/77.5.1241

[68] Finkelstein JL, Fothergill A, Krisher JT, (2021) Maternal vitamin B12 deficiency and perinatal outcomes in southern India. PLoS ONE 16, e0248145. 33822790 10.1371/journal.pone.0248145 PMC8023483

[69] Smith C (2013) Determinants of western food adoption among Hindu Nepalese women living in the Kathmandu Valley. Am J Hum Biol 25, 205–214. 23349113 10.1002/ajhb.22353

[70] Faupel-Badger JM, Hsieh CC, Troisi R, (2007) Plasma volume expansion in pregnancy: implications for biomarkers in population studies. Cancer Epidemiol Biomarkers Prev 16, 1720–1723. 17855687 10.1158/1055-9965.EPI-07-0311

