# Supplementary material for: Cobalamin and folate status in women during early pregnancy in Bhaktapur, Nepal
Source: J Nutr Sci. 2021 Aug 9;10:e57. doi: 10.1017/jns.2021.53 (PMC8358842; doi:10.1017/jns.2021.53)
Supplement: Supplementary file 1 [file S2048679021000537sup001.docx]

**Table S1:** Association between plasma cobalamin concentration and indicators of socio-economic status, and maternal and dietary characteristics among 561 pregnant women (<15 weeks of gestation) living in Bhaktapur, Nepal.

| **Variable** | **N** | **Crude**  **coefficient** | **95% CI** | **Adjusted coefficient** | **95% CI** |
| --- | --- | --- | --- | --- | --- |
| Age (years) | 561 | -1.68 | -3.39, 0.03 |  |  |
| Body Mass Index (kg/m^2^) | 561 | -0.60 | -2.74, 1.54 |  |  |
| Vegetarian mothers^†^ |  |  |  |  |  |
| Non-Vegetarian | 399 | Ref |  | Ref |  |
| Vegetarian | 7 | -69.8 | -127.3, -12.3 | -66.6 | -123.9, -9.37 |
| Gestational age (weeks)^‡^ | 561 | -2.96 | -5.23, -0.69 |  |  |
| Parity (%) |  |  |  |  |  |
| Primi | 237 | Ref |  |  |  |
| Second gravida or more | 324 | -17.1 | -30.2, -4.00 |  |  |
| Educational level (years) |  |  |  |  |  |
| Illiterate or primary level | 44 | Ref |  | Ref |  |
| Secondary level (6^th^-12^th^ grade) | 392 | 2.73 | -21.8, 27.2 | 13.2 | -14.5, 41.5 |
| Bachelor level and above | 125 | 10.5 | -16.5, 37.6 | 36.7 | 5.73, 67.7 |
| Occupation of mother |  |  |  |  |  |
| Housewife | 182 | Ref |  |  |  |
| Services | 165 | 3.49 | -13.0, 20.0 |  |  |
| Business | 116 | 2.03 | -16.2, 20.3 |  |  |
| Daily wage | 81 | -15.8 | -36.5, 4.71 |  |  |
| Others | 17 | -10.3 | -49.4, 28.8 |  |  |
| Average monthly household income, (Nepali rupees) | 561 | 0.03 | -0.09, 0.15 |  |  |
| Family type |  |  |  |  |  |
| Nuclear | 245 | Ref |  |  |  |
| Joint | 438 | 2.53 | -11.2, 16.3 |  |  |
| Household size | 561 | -0.91 | -3.48, 1.65 |  |  |
| Female head of household | 561 | -4.83 | -24.2, 14.5 |  |  |
| Ethnicity of household |  |  |  |  |  |
| Newar | 525 | Ref |  | Ref |  |
| Brahmin/ Chhetri | 71 | -29.4 | -51.6, -7.16 | -19.8 | -45.8, 6.22 |
| Gurung/Rai/Magar/Tamang | 69 | 39.7 | 17.9, 61.6 | 54.8 | 27.4, 82.2 |
| Others | 21 | -3.73 | -43.5, 36.1 | -15.3 | -62.2, 31.6 |
| Alcohol consumption^§^ |  |  |  |  |  |
| No alcohol | 309 | Ref |  |  |  |
| Once a month or less | 208 | -5.16 | -18.9, 8.66 |  |  |
| Few times a month | 38 | 1.67 | -24.8, 28.1 |  |  |
| More than once a week | 6 | 51.7 | -11.7, 115.3 |  |  |
| Pregnancy related complaints |  |  |  |  |  |
| No complaints | 450 | Ref |  | Ref |  |
| Complaints | 236 | 12.5 | -0.82, 25.9 | 22.5 | 7.39, 37.6 |
| Hemoglobin | 561 | 5.95 | 0.05, 11.8 |  |  |

Crude coefficients from bivariable linear regression analyses and adjusted regression coefficients from multivariable linear regression analyses are reported together with their corresponding 95% confidence interval (CI).

^†^ N=406, vegetarian was defined as not eating meat and eggs.

^‡^ Assessed by the last menstrual period (LMP) method and confirmed by ultrasound scan.

^§^ Usually local rice beer.

**Table S2:** Association between plasma folate concentration and indicators of socio-economic status, and maternal and dietary characteristics among 561 pregnant women (<15 weeks of gestation) living in Bhaktapur, Nepal.

| **Variable** | **N** | **Crude**  **coefficient** | **95% CI** | **Adjusted coefficient** | **95% CI** |
| --- | --- | --- | --- | --- | --- |
| Age (years) | 561 | 0.01 | 0.003,0.03 | 0.02 | 0.006, 0.03 |
| Body Mass Index (kg/m^2^) | 561 | 0.002 | -0.01, 0.02 |  |  |
| Vegetarian mothers^†^ |  |  |  |  |  |
| Non-Vegetarian | 399 | Ref |  |  |  |
| Vegetarian | 7 | -0.11 | -0.61, 0.37 |  |  |
| Gestational age (weeks)^‡^ | 561 | 0.01 | -0.001, 0.03 | 0.02 | 0.002, 0.04 |
| Parity (%) |  |  |  |  |  |
| Primi | 237 | Ref |  | Ref |  |
| Second gravida or more | 324 | -0.11 | -0.22, -0.008 | -0.18 | -0.30, -0.06 |
| Educational level (years) |  |  |  |  |  |
| Illiterate or primary level | 44 | Ref |  |  |  |
| Secondary level (6^th^-12^th^ grade) | 392 | 0.02 | -0.17, 0.22 |  |  |
| Bachelor level and above | 125 | 0.19 | -0.02, 0.41 |  |  |
| Occupation of mother |  |  |  |  |  |
| Housewife | 182 | Ref |  |  |  |
| Services | 165 | 0.07 | -0.06, 0.20 |  |  |
| Business | 116 | -0.11 | -0.26, 0.03 |  |  |
| Daily wage | 81 | -0.11 | -0.28, 0.05 |  |  |
| Others | 17 | -0.09 | -0.41, 0.22 |  |  |
| Average monthly household income, (Nepali rupees) | 561 | 0.001 | 0.0003, 0.002 | 0.001 | 0.0001, 0.002 |
| Family type |  |  |  |  |  |
| Nuclear | 245 | Ref |  |  |  |
| Joint | 438 | 0.13 | 0.02, 0.24 |  |  |
| Household size | 561 | 0.021 | 0.0007, 0.04 |  |  |
| Female head of household | 561 | 0.04 | -0.11, 0.20 |  |  |
| Ethnicity of household |  |  |  |  |  |
| Newar | 525 | Ref |  | Ref |  |
| Brahmin/ Chhetri | 71 | -0.01 | -0.20, 0.16 | 0.03 | -0.15, 0.21 |
| Gurung/Rai/Magar/Tamang | 69 | -0.34 | -0.52, -0.16 | -0.31 | -0.49, -0.13 |
| Others | 21 | -0.14 | -0.47, 0.18 | -0.11 | -0.46, 0.23 |
| Alcohol consumption^§^ |  |  |  |  |  |
| No alcohol | 309 | Ref |  |  |  |
| Once a month or less | 208 | -0.30 | -0.82, 0.22 |  |  |
| Few times a month | 38 | -0.11 | -0.33, 0.10 |  |  |
| More than once a week | 6 | 0.05 | -0.06, 0.16 |  |  |
| Pregnancy related complaints |  |  |  |  |  |
| No complaints | 450 | Ref |  |  |  |
| Complaints | 236 | 0.03 | -0.07, 0.14 |  |  |
| Hemoglobin | 561 | -0.01 | -0.06, 0.03 |  |  |

Crude coefficients from bivariable linear regression analyses and adjusted regression coefficients from multivariable linear regression analyses are reported together with their corresponding 95% confidence interval (CI).

^†^ N=406, vegetarian was defined as not eating meat and eggs.

^‡^ Assessed by the last menstrual period (LMP) method and confirmed by ultrasound scan.

^§^ Usually local rice beer.

**Table S3:** Association between plasma total homocysteine (tHcy) concentration and indicators of socio-economic status, and maternal and dietary characteristics among 561 pregnant women (<15 weeks of gestation) living in Bhaktapur, Nepal.

| **Variable** | **N** | **Crude**  **coefficient** | **95% CI** | **Adjusted coefficient** | **95% CI** |
| --- | --- | --- | --- | --- | --- |
| Age (years) | 561 | -0.005 | -0.01, 0.001 |  |  |
| Body Mass Index (kg/m^2^) | 561 | -0.006 | -0.01, 0.002 |  |  |
| Vegetarian mothers^†^ |  |  |  |  |  |
| Non-Vegetarian | 399 | Ref |  | Ref |  |
| Vegetarian | 7 | 0.54 | 0.32, 0.77 | 0.57 | 0.35, 0.79 |
| Gestational age (weeks)^‡^ | 561 | -0.01 | -0.02, -0.008 | -0.02 | -0.03, -0.01 |
| Parity (%) |  |  |  |  |  |
| Primi | 237 | Ref |  |  |  |
| Second gravida or more | 324 | -0.01 | -0.06, 0.03 |  |  |
| Educational level (years) |  |  |  |  |  |
| Illiterate or primary level | 44 | Ref |  | Ref |  |
| Secondary level (6^th^-12^th^ grade) | 392 | -0.03 | -0.13, 0.05 | -0.02 | -0.13, 0.07 |
| Bachelor level and above | 125 | -0.10 | -0.21, 0.001 | -0.12 | -0.24, -0.01 |
| Occupation of mother |  |  |  |  |  |
| Housewife | 182 | Ref |  |  |  |
| Services | 165 | -0.004 | -0.069, 0.061 |  |  |
| Business | 116 | -0.015 | -0.08, 0.05 |  |  |
| Daily wage | 81 | 0.05 | -0.03, 0.13 |  |  |
| Others | 17 | 0.06 | -0.09, 0.21 |  |  |
| Average monthly household income, (Nepali rupees) | 561 | -0.000 | -0.0005, 0.0004 |  |  |
| Family type |  |  |  |  |  |
| Nuclear | 245 | Ref |  |  |  |
| Joint | 438 | -0.006 | -0.06, 0.04 |  |  |
| Household size | 561 | 0.001 | -0.008, 0.01 |  |  |
| Female head of household | 561 | 0.04 | -0.03, 0.11 |  |  |
| Ethnicity of household |  |  |  |  |  |
| Newar | 525 | Ref |  |  |  |
| Brahmin/ Chhetri | 71 | 0.11 | 0.03, 0.20 |  |  |
| Gurung/Rai/Magar/Tamang | 69 | 0.09 | 0.004, 0.17 |  |  |
| Others | 21 | -0.07 | -0.23, 0.07 |  |  |
| Alcohol consumption^§^ |  |  |  |  |  |
| No alcohol | 309 | Ref |  |  |  |
| Once a month or less | 208 | -0.04 | -0.10, 0.004 |  |  |
| Few times a month | 38 | -0.01 | -0.11, 0.09 |  |  |
| More than once a week | 6 | 0.06 | -0.18, 0.31 |  |  |
| Pregnancy related complaints |  |  |  |  |  |
| No complaints | 450 | Ref |  |  |  |
| Complaints | 236 | 0.01 | -0.04, 0.06 |  |  |
| Hemoglobin | 561 | 0.007 | -0.01, 0.03 |  |  |

Crude coefficients from bivariable linear regression analyses and adjusted regression coefficients from multivariable linear regression analyses are reported together with their corresponding 95% confidence interval (CI).

^†^ N=406, vegetarian was defined as not eating meat and eggs

^‡^ Assessed by the last menstrual period (LMP) method and confirmed by ultrasound scan

^§^ Usually local rice beer

**Table S4:** Association between plasma methylmalonic acid (MMA) concentration and indicators of socio-economic status, and maternal and dietary characteristics among 561 pregnant women (<15 weeks of gestation) living in Bhaktapur, Nepal.

| **Variable** | **N** | **Crude**  **coefficient** | **95% CI** | **Adjusted coefficient** | **95% CI** |
| --- | --- | --- | --- | --- | --- |
| Age (years) | 561 | 0.006 | -0.006, 0.018 |  |  |
| Body Mass Index (kg/m^2^) | 561 | -0.02 | -0.03, -0.008 | -0.01 | -0.03, -0.001 |
| Vegetarian mothers^†^ |  |  |  |  |  |
| Non-Vegetarian | 399 | Ref |  | Ref |  |
| Vegetarian | 7 | 0.49 | 0.07, 0.92 | 0.48 | 0.04, 0.92 |
| Gestational age (weeks)^‡^ | 561 | 0.017 | 0.0007, 0.03 |  |  |
| Parity (%) |  |  |  |  |  |
| Primi | 237 | Ref |  |  |  |
| Second gravida or more | 324 | -0.02 | -0.12, 0.06 |  |  |
| Educational level (years) |  |  |  |  |  |
| Illiterate or primary level | 44 | Ref |  |  |  |
| Secondary level (6^th^-12^th^ grade) | 392 | -0.01 | -0.18, 0.16 |  |  |
| Bachelor level and above | 125 | -0.02 | -0.22, 0.16 |  |  |
| Occupation of mother |  |  |  |  |  |
| Housewife | 182 | Ref |  | Ref |  |
| Services | 165 | 0.06 | -0.05, 0.18 | 0.06 | -0.08, 0.20 |
| Business | 116 | 0.01 | -0.11, 0.14 | 0.08 | -0.07, 0.23 |
| Daily wage | 81 | 0.20 | 0.06, 0.35 | 0.21 | 0.04, 0.39 |
| Others | 17 | 0.18 | -0.09, 0.46 | 0.18 | -0.17, 0.55 |
| Average monthly household income, (Nepali rupees) | 561 | 0.0001 | -0.001, 0.001 |  |  |
| Family type |  |  |  |  |  |
| Nuclear | 245 | Ref |  |  |  |
| Joint | 438 | -0.01 | -0.11, 0.08 |  |  |
| Household size | 561 | 0.01 | -0.008, 0.02 |  |  |
| Female head of household | 561 | 0.02 | -0.11, 0.16 |  |  |
| Ethnicity of household |  |  |  |  |  |
| Newar | 525 | Ref |  | Ref |  |
| Brahmin/ Chhetri | 71 | 0.21 | -0.04, 0.28 | 0.06 | -0.13, 0.25 |
| Gurung/Rai/Magar/Tamang | 69 | -0.24 | -0.40, -0.08 | -0.30 | -0.50, -0.10 |
| Others | 21 | -0.02 | -0.31, 0.26 | 0.02 | -0.33, 0.37 |
| Alcohol consumption^§^ |  |  |  |  |  |
| No alcohol | 309 | Ref |  |  |  |
| Once a month or less | 208 | 0.001 | -0.09, 0.10 |  |  |
| Few times a month | 38 | 0.05 | -0.13, 0.25 |  |  |
| More than once a week | 6 | 0.04 | -0.42, 0.50 |  |  |
| Pregnancy related complaints |  |  |  |  |  |
| No complaints | 450 | Ref |  |  |  |
| Complaints | 236 | -0.07 | -0.17, 0.02 |  |  |
| Hemoglobin | 561 | -0.04 | -0.08, -0.001 |  |  |

Crude coefficients from bivariable linear regression analyses and adjusted regression coefficients from multivariable linear regression analyses are reported together with their corresponding 95% confidence interval (CI).

^†^ N=406, vegetarian was defined as not eating meat and eggs

^‡^ Assessed by the last menstrual period (LMP) method and confirmed by ultrasound scan

^§^ Usually local rice beer

**Table S5:** Association between a combined vitamin B12 indicator (3cB12) and indicators of socio-economic status, and maternal and dietary characteristics among 561 pregnant women (<15 weeks of gestation) living in Bhaktapur, Nepal.

| **Variable** | **N** | **Crude**  **coefficient** | **95% CI** | **Adjusted coefficient** | **95% CI** |
| --- | --- | --- | --- | --- | --- |
| Age (years) | 561 | 0.001 | -0.012, 0.013 |  |  |
| Body Mass Index (kg/m^2^) | 561 | 0.01 | 0.002, 0.03 |  |  |
| Vegetarian mothers^†^ |  |  |  |  |  |
| Non-Vegetarian | 399 | Ref |  | Ref |  |
| Vegetarian | 7 | -0.89 | -1.34, -0.45 | -0.90 | -1.36, -0.44 |
| Gestational age (weeks)^‡^ | 561 | -0.008 | -0.02, 0.009 |  |  |
| Parity (%) |  |  |  |  |  |
| Primi | 237 | Ref |  |  |  |
| Second gravida or more | 324 | 0.007 | -0.09, 0.10 |  |  |
| Educational level (years) |  |  |  |  |  |
| Illiterate or primary level | 44 | Ref |  | Ref |  |
| Secondary level (6^th^-12^th^ grade) | 392 | 0.02 | -0.16, 0.21 | 0.11 | -0.11, 0.33 |
| Bachelor level and above | 125 | 0.08 | -0.11, 0.29 | 0.29 | 0.03, 0.55 |
| Occupation of mother |  |  |  |  |  |
| Housewife | 182 | Ref |  | Ref |  |
| Services | 165 | -0.02 | -0.15, 0.10 | -0.13 | -0.29, 0.02 |
| Business | 116 | 0.01 | -0.12, 0.15 | -0.08 | -0.23, 0.07 |
| Daily wage | 81 | -0.19 | -0.35, -0.03 | -0.21 | -0.39, -0.03 |
| Others | 17 | -0.17 | -0.47, 0.12 | -0.21 | -0.59, 0.16 |
| Average monthly household income, (Nepali rupees) | 561 | -0.000 | -0.0005, 0.0004 |  |  |
| Family type |  |  |  |  |  |
| Nuclear | 245 | Ref |  |  |  |
| Joint | 438 | 0.01 | -0.09, 0.11 |  |  |
| Household size | 561 | -0.009 | -0.02, 0.01 |  |  |
| Female head of household | 561 | -0.05 | -0.20, 0.09 |  |  |
| Ethnicity of household |  |  |  |  |  |
| Newar | 525 | Ref |  | Ref |  |
| Brahmin/ Chhetri | 71 | -0.26 | -0.44, -0.09 | -0.17 | -0.37, 0.03 |
| Gurung/Rai/Magar/Tamang | 69 | 0.18 | 0.01, 0.35 | 0.31 | 0.09, 0.53 |
| Others | 21 | 0.01 | -0.29, 0.32 | -0.07 | -0.44, 0.29 |
| Alcohol consumption^§^ |  |  |  |  |  |
| No alcohol | 309 | Ref |  |  |  |
| Once a month or less | 208 | 0.02 | -0.08, 0.13 |  |  |
| Few times a month | 38 | -0.04 | -0.24, 0.16 |  |  |
| More than once a week | 6 | 0.12 | -0.36, 0.61 |  |  |
| Pregnancy related complaints |  |  |  |  |  |
| No complaints | 450 | Ref |  |  |  |
| Complaints | 236 | 0.06 | -0.03, 0.17 |  |  |
| Hemoglobin | 561 | 0.04 | -0.003, 0.08 |  |  |

Crude coefficients from bivariable linear regression analyses and adjusted regression coefficients from multivariable linear regression analyses are reported together with their corresponding 95% confidence interval (CI).

^†^ N=406, vegetarian was defined as not eating meat and eggs.

^‡^ Assessed by the last menstrual period (LMP) method and confirmed by ultrasound scan.

^§^ Usually local rice beer.
